# Damage to the *Drosophila* follicle cell epithelium produces “false clones” with apparent polarity phenotypes

**DOI:** 10.1242/bio.20134671

**Published:** 2013-09-23

**Authors:** Timm Haack, Dan T. Bergstralh, Daniel St Johnston

**Affiliations:** The Gurdon Institute and the Department of Genetics, University of Cambridge, Tennis Court Road, Cambridge CB2 1QN, UK

**Keywords:** *Drosophila*, False clones, Follicle cell epithelia, Polarity

## Abstract

The *Drosophila* follicular epithelium, which surrounds developing egg chambers, is a well-established model for studying epithelial polarity because it is continuously generated from adult stem cells, making it easy to generate homozygous mutant clones in a heterozygous background. Mutant clones are usually marked by the loss of Green Fluorescent Protein (GFP) expression, which distinguishes them from their green, wild-type neighbours. Here we report that damage to the epithelium during dissection can produce groups of GFP-negative cells that resemble mutant clones. Furthermore, several polarity factors, such as aPKC and Discs large, are not localised in these damage-induced false clones. This phenotype is identical to that reported for several mutants, including *ampk* and *Dystroglycan* mutant clones under conditions of energetic stress. Using more reliable systems to mark *ampk* and *Dystroglycan* null clones such as the MARCM system, we found that neither protein is required for epithelial polarity under low energy conditions. Thus, our previous report of a specific low energy polarity pathway is an artefact of the increased damage caused by dissecting the small ovaries of starved flies. However, *ampk* mutant cells are larger than normal under both starvation and well-fed conditions, indicating that AMPK restricts follicle cell growth even when dietary sugar is not limiting. We suspect that several other reports of mutants that disrupt follicle cell polarity may also be based on the phenotype of damage-induced false clones, and recommend the use of positively marked clones to avoid this potential artefact.

## Introduction

Epithelia are sheets of cells that serve as barriers between different compartments in the body and are also responsible for the directed transport of molecules. To fulfil their function, epithelial cells need to polarise along their apical–basal axis and this process is controlled by a set of conserved polarity proteins localising to distinct membrane domains. Most malignant tumours arise from epithelial tissues and loss of polarity is a hallmark of the epithelial–mesenchymal transition, a critical step in cancer metastasis. Hence, the question of how epithelia establish and maintain polarisation has been intensely studied in different systems. Included among these is the *Drosophila* follicular epithelium.

The follicle cells ensheath the germline cyst of 15 nurse cells and an oocyte that ultimately develops into the egg (Bastock and St Johnston, 2008). As a secondary epithelium, follicle cells arise from somatic stem cells in the germarium and undergo polarisation through a mesenchymal–epithelial transition. Apical–basal polarity of follicle cells is defined by a set of conserved polarity proteins that define distinct membrane domains (St Johnston and Ahringer, 2010). The apical domain facing the germline is characterised by the transmembrane protein Crumbs, atypical protein kinase C (aPKC) and Par-6, whereas the apical/lateral junction is defined by adherens junctions, which are positioned by Bazooka (Par-3 in other organisms) (Benton and St Johnston, 2003; Morais-de-Sá et al., 2010; Tanentzapf et al., 2000). Scribble, Discs large (Dlg), and Lethal (2) giant larvae (Lgl) all localise to the lateral membrane, where they antagonise apical factors (Bilder et al., 2000). The organisation of the follicle cell microtubule cytoskeleton relies on Par-1, which is also lateral (Doerflinger et al., 2003). The basal surface is characterised by integrins and the transmembrane glycoprotein Dystroglycan (Dg) linking the extracellular matrix with the actin cytoskeleton (St Johnston and Ahringer, 2010).

Follicle cells are easily imaged, exist within the context of a tissue rather than a cultured system, and may be genetically manipulated via mitotic recombination to produce homozygous mutant clones within an otherwise wild-type tissue. The latter feature allows for a side-by-side comparison of mutant and wild-type cells; cells homozygous for a mutant allele of interest are typically marked by the absence of GFP. Such comparisons have yielded important insights into the establishment and maintenance of epithelial polarity as well as the consequences of its breakdown (Benton and St Johnston, 2003; Bilder et al., 2000; Morais-de-Sá et al., 2010; Tanentzapf et al., 2000; and others).

Recent work from the St Johnston laboratory demonstrated the existence of a distinct polarisation pathway in the follicle cells that is only required under conditions of energetic stress. This low-energy polarity pathway comprises activation of the AMP-activated protein kinase AMPK by the serine/threonine kinase LKB1 and a basal cue provided by the extracellular matrix component Perlecan and its receptor Dystroglycan (Mirouse et al., 2009; Mirouse et al., 2007). In following up on these findings, we observed that AMPK controls cell size in the follicle cell epithelium, but were unable to reproduce the energy-dependent polarity phenotype. We further show that the reported low-energy polarity mutants do not lose polarity under starvation conditions and that the phenotype observed is due to a damage artefact, which may explain other reports of polarity phenotypes in the literature.

## Results

### *ampk* and *Dg* mutant cells retain polarity under starvation conditions

**Fig. 1.**
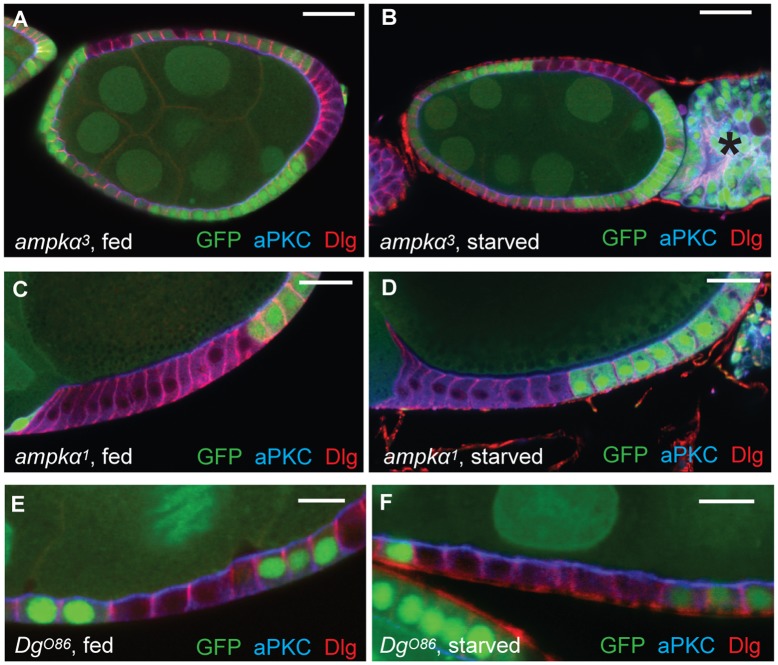
***ampk* and *Dg* mutant cells do not lose apical–basal polarity under starvation conditions.** (**A**,**B**) A well-fed early stage 8 egg chamber (A) and a starved stage 7 egg chamber (B) containing *ampkα^3^* follicle cell clones marked by the absence of GFP. Starved *ampkα^3^* mutant cells show normal localisation of the apical polarity determinant, aPKC (blue), and the lateral polarity factor, Dlg (red). (**C**,**D**) A well-fed (C) and a starved (D) stage 10 egg chamber containing *ampkα^1^* clones marked by the absence of GFP. aPKC (blue) and Dlg (red) localise normally in both well-fed and starved mutant clones. (**E**,**F**) Well-fed (E) and starved (F) stage 8 egg chambers containing *Dg^O86^* clones marked by the absence of GFP. Starved *Dg^O86^* clones show normal localisation of aPKC (blue) and Dlg (red). Note the presence of dying egg chambers (B, asterisk) that are frequently observed in nutrient-deprived ovarioles. Scale bars: 25 µm (A–D), 10 µm (E,F).

To analyse the role of AMPK in epithelial polarity in more detail, we generated homozygous clones of *ampkα^3^* and *ampkα^1^* using the Flp/FRT system in which the loss of GFP-nls marks mutant clones. The homozygous mutant cells showed normal polarity under both well-fed and starvation conditions, as indicated by the regular epithelial architecture and the wild-type localisation of Dlg and aPKC (Fig. 1A–D). The Dystroglycan allele *Dg^O86^* has also been reported to cause a starvation-dependent loss of epithelial polarity. However, like the *ampk* alleles, large *Dg^O86^* clones maintained normal apical–basal polarity in our hands (Fig. 1E,F). We used the same starvation protocol as previously described (Mirouse et al., 2007) and all mutant stocks were confirmed by sequencing (supplementary material Fig. S1). One possible explanation for the lack of a phenotype in these experiments is that the starvation protocol did not induce sufficient energetic stress to activate the low energy polarity pathway. We therefore attempted to increase the energetic stress in the follicle cells by feeding flies drugs that reduce cellular ATP levels and activate AMPK, including 2-deoxyglucose (a glycolysis inhibitor), Oligomycin (an inhibitor of mitochondrial ATP synthase), Metformin (an inhibitor of mitochondrial complex I), tetracycline and chloramphenicol (inhibitors of mitochondrial protein synthesis), berberine (an AMPK activator that is thought to inhibit mitochondrial respiration) and paraquat (an inducer of oxidative stress) (Bus and Gibson, 1984; Cohen and Saneto, 2012; Hawley et al., 2010). *Dg* and *ampk* mutant clones still showed normal polarity after treatment with these drugs, though many egg chambers degenerated at stage 7/8 because drug-induced starvation activated a nutritional checkpoint that blocks vitellogenesis (Drummond-Barbosa and Spradling, 2001) (supplementary material Table S1). These negative results strongly suggest that AMPK and Dystroglycan are not required for apical–basal polarity under starvation conditions in the follicle cell epithelium.

### Mechanical damage induces pseudo-clones that mimic polarity phenotypes

**Fig. 2.**
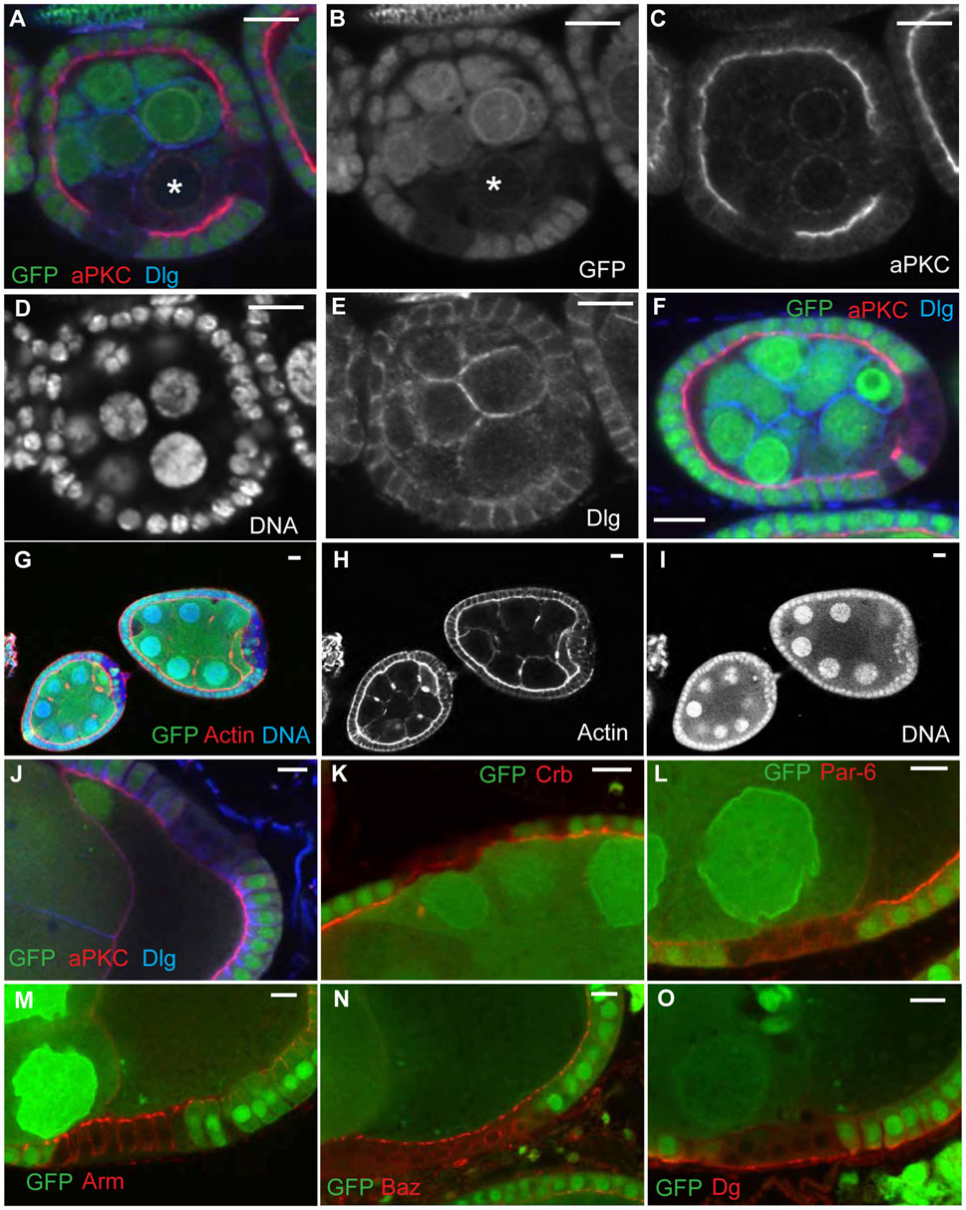
Damage to follicle cells produces false clones that lose polarity markers. Egg chambers from *FRTG13 GFPnls/CyO* females damaged by pipetting before fixation contain false clones indicated by the loss of nuclear GFP. (**A–E**) A stage 4 egg chamber containing two damage-induced false clones that have lost nuclear GFP, showing the concomitant loss of aPKC (C) and Dlg (E) staining. Note that the nurse cells adjacent to the damaged follicle cells also show reduced nuclear GFP signal (asterisk, A,B). The damaged epithelium maintains overall integrity indicated by DAPI staining of follicle cell nuclei (D). (**F**) A damage-induced false clone at the posterior of a stage 4 egg chamber showing the loss of polarity markers. Note that GFP can be lost from only the follicle cells without affecting the signal in adjacent germline cells (compare A and F). (**G–I**) Damage-induced false clones that display multilayering at the posterior of the egg chamber, with a loss of basal actin (H) and disorganisation of the epithelium (I). (**J**) A stage 9 egg chamber showing loss of aPKC and Dlg in a false clone. (**K**) A stage 8 egg chamber with a damage-induced false clone stained for Crb. The Crb signal is almost completely lost from the apical plasma membrane in damaged follicle cells. (**L**) A stage 9 egg chamber with a damage-induced false clone stained for Par-6. The Par-6 signal is completely lost from damaged follicle cells. (**M**) Stage 9 egg chamber stained for Arm shows that adherens junctions persist in damaged follicle cells. (**N**) A stage 9 egg chamber stained for Baz. Baz localisation is not affected in damaged follicle cells. (**O**) A damage-induced false clone in a stage 9 egg chamber stained for Dystroglycan (Dg). Dg signal is not lost from the basal side of damaged follicle cells. Scale bars: 10 µm.

In the experiments described above, we observed a low frequency of “clones” that had lost both GFP and cortical polarity markers, but closer examination revealed that the adjacent nurse cells also showed reduced GFP expression. As germline cells should not be affected by mitotic recombination in the follicular epithelium, we were concerned that the GFP signal had disappeared for some other reason, and one possible explanation was mechanical damage. In a standard laboratory protocol, ovaries are dissociated into ovarioles prior to immunostaining by pipetting the sample up and down several times, typically with a 1000 µl tip. We hypothesised that mechanical damage to the follicle epithelium caused by this pipetting caused some follicle cells, as well as adjacent nurse cells, to concomitantly lose GFP and polarity factors. To test this hypothesis, we pipetted ovaries from a *Drosophila* line expressing GFP-nls (*FRTG13 GFPnls/CyO*) up and down with a 200 µl pipette to cause mechanical damage before fixation. Since these flies carry only a single FRT site and no source of the Flp recombinase, the GFP signal cannot be lost as a result of mitotic recombination in this genotype. Nevertheless, this treatment caused groups of follicle cells to lose the GFP signal. Furthermore, the GFP-negative cells also lost their staining for several polarity markers, including aPKC and Dlg, thereby mimicking a GFP-negative mutant clone with a polarity phenotype. We will refer to these GFP-negative regions of damaged follicle cells as “false clones”, as they resemble the appearance of true genetic clones (Fig. 2A–F). False clones were also found in experiments in which samples were fixed before pipetting, albeit at a lower frequency. The loss of GFP from damaged follicle cells can be incomplete, which offers a way to distinguish them from real clones. Damaged regions may also be characterised by a decrease in basal actin, a disorganised “multi-layered” appearance of cells, and in severe cases, the separation of the follicle cell layer from the basement membrane (Fig. 2G–I).

The loss of GFP and polarity markers in false clones does not require substantial damage to the egg chamber, as the overall organisation of the follicle cell layer is unaffected in most cases (Fig. 2D). This may be because clones of follicle cells remain interconnected after mitosis by cytoplasmic bridges called ring canals that allow cytoplasmic proteins to move between cells (Airoldi et al., 2011). This therefore suggests that damage to one cell can lead to the loss of GFP from multiple cells. In addition, although germline cells adjacent to false clones may lose GFP, they are not always affected, making it harder to discern a real from a false clone in this way (Fig. 2F,K,L).

We characterised the phenotype of damage-induced false clones in more detail by staining for other polarity factors. Like aPKC, the apical proteins, Crumbs (Crb) and Par-6 are lost from damaged follicle cells (Fig. 2K,L). By contrast, the localisation of the adherens junction components Armadillo (Arm) and Bazooka (Baz) is not obviously affected (Fig. 2M,N). Staining for the extracellular matrix receptor Dystroglycan is also maintained, indicating that the integrity of the basal surface is not severely disrupted in false clones (Fig. 2O). In rare cases, damaged follicle cells show an increase in Dg staining, which extends around the lateral and apical membrane domains (supplementary material Fig. S2K,L). This may be due to the increased penetration of the fixative or the primary and secondary antibodies into damaged cell clones, allowing better detection of Dg deeper inside the sample. The lateral proteins Fasciclin 3 (FasIII) and Coracle (Cora) seem to be resistant to damage-induced loss in false clones, although the FasIII signal is sometimes decreased in the damaged area (supplementary material Fig. S2). By contrast, the staining for βH-spectrin is increased in damaged follicle cells. If this increase is because of improved antibody penetration it reveals that the protein is present at low levels on the lateral and basal cortex, as well as apically (supplementary material Fig. S2).

The phenotypes of damage-induced false clones closely resemble those reported for *ampk* and *Dg* mutant cells under conditions of energetic stress: apical (Crb, Par-6, aPKC) and lateral (Dlg) markers disappear, whereas adherens junction components (Arm, Baz) remain localised and Dystroglycan apparently extends around the cortex. Given our failure to reproduce the published phenotypes under even more stringent starvation conditions, we conclude that the reported function of AMPK and Dg in epithelial polarity under energetic stress is actually a misinterpretation of damage-induced false clones that mimic a loss of apical–basal polarity.

**Fig. 3.**
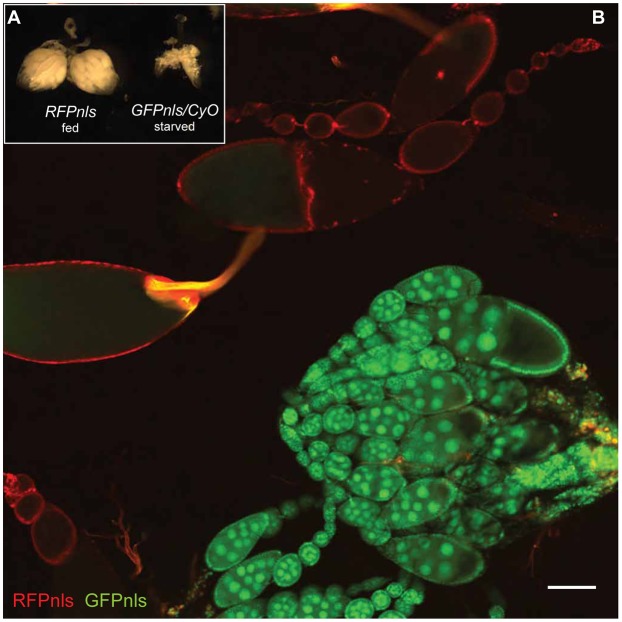
Ovarioles of starved ovaries are resistant to separation by pipetting. Ovaries from either well-fed (*RFPnls*) females or females starved on low-sugar medium (*GFPnls/CyO*) for 2 days. (**A**) Starved ovaries are reduced in size compared to well-fed ovaries. (**B**) Ovaries of both populations were dissected in PBT (PBS 0.2% Tween 20), mixed, and the ovarioles separated by pipetting 10× with a 200 µl tip before fixation. Whilst ovarioles from well-fed flies separate easily, the starved ovarioles remain stuck together in big clusters. Scale bar: 100 µm.

A question arises from these results: why did *ampk* and *Dg* mutants only appear to give polarity phenotypes under starvation conditions? Nutrient deprivation in flies leads to a reduction in ovary size (Fig. 3A), making it more difficult to separate them into ovarioles during sample preparation. To demonstrate this, we combined ovaries from well-fed flies expressing RFP-nls and ovaries from starved flies expressing GFP-nls and pipetted them up-and-down ten times in a 200 µl tip. Well-fed ovaries separated easily into individual ovarioles whilst starved ovaries remained in clusters (Fig. 3B). This indicates that more force is required to separate starved ovaries, and may explain why damaged-induced false clones are much more frequent in these samples, giving the erroneous impression that the “mutant” phenotype is starvation-dependent.

### Other reported polarity phenotypes may be a consequence of egg chamber damage

**Fig. 4.**
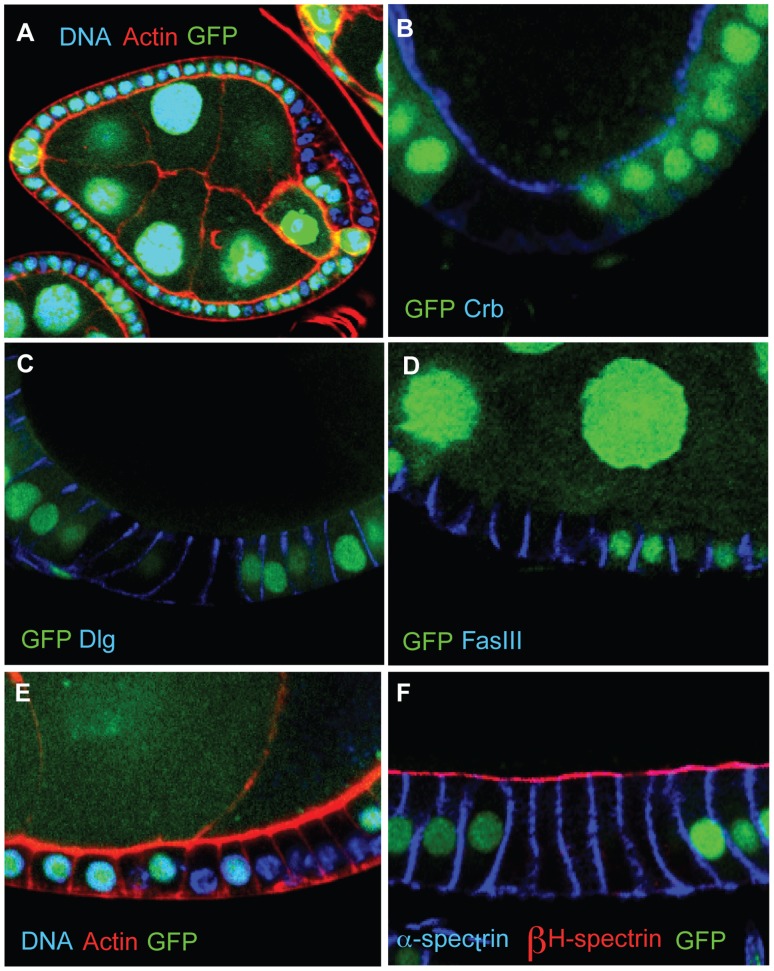
***pak* mutant clones do not lose apical–basal polarity.** (**A**) A *pak^14^* mutant clone marked by the loss of GFP producing a multi-layered epithelium at the posterior of the egg chamber, as previously reported. (**B–D**) Expression and localisation of the polarity factors, Crb, Dlg, and FasIII, are unchanged in *pak^14^* mutant clones (marked by the absence of GFP). (**E**) Basal actin is normal in *pak^14^* mutant clones. (**F**) β-Heavy-spectrin is unchanged in *pak^14^* mutant clones.

Having characterised the artefact, we re-examined other publications from the lab and discovered two that may contain further examples of damage-induced false clones. Figure 1A in Doerflinger et al. shows a *par-1*^W3^ clone with no apical aPKC staining that resembles a damaged-induced clone, and shows a phenotype that was not observed in most small clones (Doerflinger et al., 2003). We therefore re-analysed the original data and observed a number of bona fide *par-1* clones, as judged by the presence of twin-spot clones and the absence of residual GFP signal. Although most clones show normal aPKC localisation, a significant proportion has reduced levels of apical aPKC and a few lack apical aPKC entirely, and the original conclusions of this paper are therefore unchanged. Secondly, figure 4b of Martin and St Johnston shows an *lkb1* mutant clone with reduced apical aPKC signal (Martin and St Johnston, 2003), which is a hallmark of the damage-induced phenotype. Nevertheless, the other panels in this figure used an approach that does not involve negatively marked clones to show that the removal of LKB1 from the entire follicle cell lineage disrupts the epithelium. Thus, the panels containing these possible “false” clones are peripheral to the main conclusions of both papers, which are unaffected by the possible artefact.

Several other reports in the literature also describe phenotypes that resemble those of damage-induced false clones. For example, follicle cell clones mutant for the Pak serine–threonine kinase have been reported to lose apical and lateral markers (Crb, Dlg and FasIII) and basal F-actin, have reduced staining for adherens junctional markers, but increased staining for β-Heavy-spectrin (Conder et al., 2007). To test whether these observations might also be due to damage-induced false clones, we generated homozygous mutant clones of the strong allele, *pak*^14^, taking particular care to avoid damaging the egg chambers. The mutant clones showed a penetrant epithelial disorganisation phenotype, as previously reported, but we observed no detectable changes in the localisation of polarity makers or basal actin (Fig. 4). Close examination of the earlier data reveals hallmarks of egg chamber damage, including incomplete loss of GFP from the alleged clone and loss of GFP from adjacent nurse cells. Thus, although the loss of Pak disrupts the organisation of the follicular epithelium, this phenotype is not caused by a defect in the apical–basal polarity of the mutant cells.

### Use of positively marked clones prevents misinterpretation of damage artefacts

**Fig. 5.**
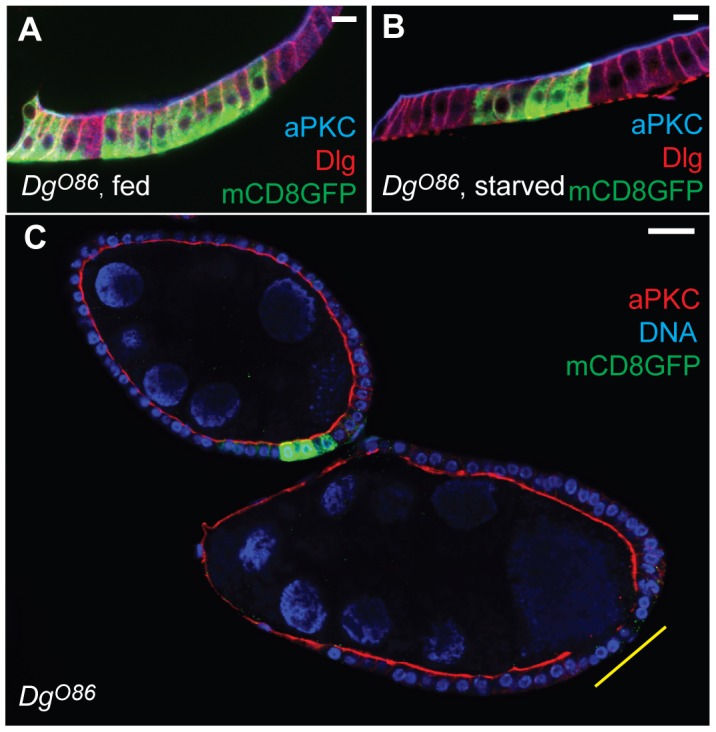
Positively marked clones are easily distinguished from damage-induced false clones. *Dg^O86^* mutant clones marked by mCD8GFP expression using the MARCM system. (**A**,**B**) Well-fed (A) and starved (B) *Dg^O86^* clones maintain proper polarisation as shown by the normal localisation of aPKC (blue) and Dlg (red). Scale bars: 10 µm. (**C**) A damage-induced false clone (lower egg chamber, yellow line) and a genuine *Dg^O86^* clone (upper egg chamber) in an ovariole that was damaged during sample preparation. Note that the aPKC signal is only lost in the damaged follicle cells. Scale bar: 20 µm.

The difficulty in distinguishing between damage-induced false clones and real clones arises because the clones are negatively marked by the absence of GFP signal. This problem can therefore be avoided by using a system in which mutant clones are marked the presence of a fluorescent marker, such as the Mosaic Analysis with a Repressible Cell Marker (MARCM) system (Lee and Luo, 1999). To confirm this, we used the MARCM system to generate *Dg^O86^* clones in well-fed and starved ovaries that were deliberately damaged by vigorous pipetting. *Dg^O86^* clones positively marked by mCD8GFP expression did not show a polarity phenotype under either condition, but false damage-induced clones were still observed (Fig. 5A–C). Because the damaged cells do not express the clonal marker, there is no risk of misclassifying these as mutant cells.

### *ampk* mutant follicle cells are larger than wild type

**Fig. 6.**
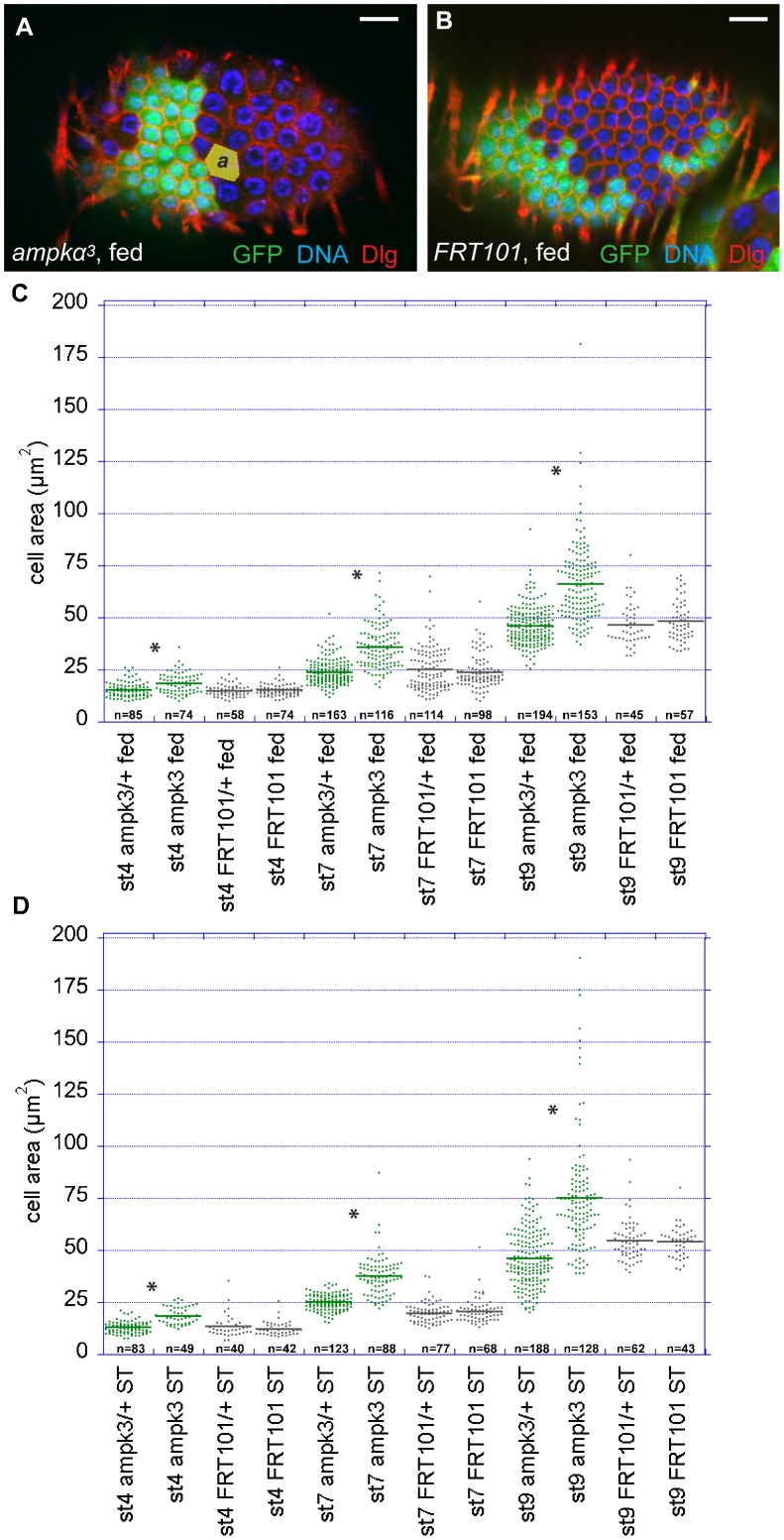
***ampkα^3^* mutant cells are larger than normal.** (**A**) A well-fed egg chamber containing an *ampkα^3^* clone marked by the absence of GFP. *ampkα*^3^ mutant cells have an increased cell area compared to the neighbouring GFP-positive *ampkα*^3^/+ cells. Scale bar: 10 µm. (**B**) A control wild-type clone marked by the absence of GFP. Cells lacking GFP are the same size as their GFP-positive neighbours. Scale bar: 10 µm. (**C**,**D**) Cell area measurements in different genotypes. Anti-Dlg immunofluorescence signal was used to mark cell outlines. The cell area (*a* in panel A) was determined using ImageJ for *ampkα^3^/+*, *ampkα^3^*, *FRT101/+* and *FRT101* cells at different stages of egg chamber development under well-fed (C, fed) and starvation (D, ST) conditions. *ampkα^3^* is denoted as “ampk3”. The mean cell area is depicted by horizontal bars. Statistical significance was calculated using the Student's t-test (*P<0.0001). n = total number of cells.

The demonstration that AMPK plays no role in the maintenance of epithelial polarity under energetic stress raises the question of whether this kinase has any function in the follicle cell layer. During our initial experiments, we noticed that cells mutant for *ampkα^1^* or *ampkα^3^* are larger than the adjacent wild-type control cells, even under normal feeding conditions (Fig. 6A,B). We therefore quantified the cell size of *ampkα^3^* mutant cells and compared them to their *ampkα*^3^/+ neighbours by measuring individual cells and calculating the mean cell area of each population at different stages of egg chamber development under well-fed and starvation conditions (Fig. 6C,D). Well-fed *ampkα^3^* cells in early egg chambers (stage 4) show an increase in mean cell area of 20% compared to *ampkα^3^/+* cells in the same tissue, and this increases after the switch from mitosis to the endocycle to 51% at stage 7 and 44% at stage 9. To rule out the possibility that this difference occurs because cells expressing GFP have a growth disadvantage, we also generated control clones with a *FRT* chromosome without the *ampk* mutation (*FRT101*). Well-fed *FRT101* cells show no significant difference in mean cell area compared to GFP-positive *FRT101/+* cells at any stage examined.

Under starvation conditions, the cell area of stage 4 *ampkα^3^* mutant cells is 45% larger than that of *ampkα^3^/+* cells. This difference is greater than under well-fed conditions (20%), but is mainly due to the smaller cell size in starved *ampkα^3^/+* control cells, as starved *ampkα^3^* mutant cells are about the same size as well-fed *ampkα^3^* cells at stage 4 (Fig. 6; supplementary material Table S2). Stage 7 *ampkα^3^* mutant cells show the same increase in cell size as in well-fed conditions, with a mean cell area 51% larger than that of control cells. At stage 9, however, starved *ampkα^3^* mutant cells were 63% larger than the control cells, which is a higher increase than in well-fed *ampkα^3^* mutant cells at the same stage (44%) (Fig. 6; supplementary material Table S2). Cumulatively, these data suggest that AMPK is already active under well-fed conditions and controls follicle cell size in normal egg chamber development.

## Discussion

The *Drosophila* follicle cell epithelium is a popular model system for the study of epithelial cell polarity. We have described an easily-produced artefact, caused by damage to the sample during preparation, that can and has been mistaken for a follicle cell polarity defect. Mutant clones generated by mitotic recombination are typically marked by the absence of GFP, and damaged cells also lose GFP, which can lead them to be mistaken for mutant clones. As damaged cells lose certain polarity factors, such as aPKC and Discs large, they have been interpreted as showing a polarity defect caused by the mutation. It is worth noting that false clones can maintain their overall architecture, and usually remain within the follicle cell monolayer. This feature contributes to the confusion caused by the artefact; apart from the apparent polarity defect, the cells often appear healthy and normal, and are thus easily mistaken for clones. The similarity between false clones and genuine clones is exacerbated by the interconnected nature of the follicle cells. Even lesions in a single cell can affect a cluster of cells, as the intercellular ring canals between follicle cells allow GFP and other proteins to leak from neighboring unaffected cells into the damaged cell (Airoldi et al., 2011; Woodruff and Tilney, 1998).

While damage is most likely to occur when egg chambers are subjected to heavy pipetting or the use of 200 µl rather than 1000 µl tips, we have also seen them in carefully prepared samples. To reduce the frequency of damaged follicle cells, we strongly advise against pipetting ovaries prior to fixation. Instead, one should carefully separate ovarioles using dissection needles and, if necessary, further separate the egg chambers after fixation using 1000 µl tips. Damaged areas can sometimes, but not always, be distinguished from genuine clones by the incomplete loss of GFP and/or the loss of GFP from the neighbouring germline cell. In tissues with genuine mutant clones, twin spot clones, which inherited two copies of GFP in the course of mitotic recombination, can often be identified by intense GFP signal in the vicinity of the GFP-negative mutant clone. Therefore, the lack of twin spots in an affected tissue suggests the presence of a damage-induced false clone rather than a genuine mutant clone. The problem of false clones can be avoided by using positive marking systems, in which mutant clones are identified by the presence rather than absence of fluorescence.

Our results indicate that two articles from the St Johnston lab showing that apical–basal polarity is lost in starved *ampk* and *Dystroglycan* mutant clones are incorrect, as the conclusions were based almost entirely on damage-induced false clones. We no longer have any evidence to support the existence of the proposed low energy polarity pathway in follicle cells. This has no bearing on the observation that *ampk* germline clones disrupt epithelial organisation in the embryonic ectoderm, as these experiments should not be susceptible to damage-induced false clones (Lee et al., 2007). A review of the field does suggest, however, that the artefact we have identified may not be uncommon. Results very similar in appearance to the reported low-energy *ampk* and *Dystroglycan* phenotype were also reported in cells mutant for the kinase *pak* (Conder et al., 2007). However, a loss of polarity factor staining is not observed in *pak* mutant clones when samples are prepared using our revised protocol, indicating that the previous observations were probably the result of damage to the egg chamber. In another recently published study, the authors noted concomitant loss of aPKC staining and GFP from follicle cells under rare circumstances in which mitotic clones had not been induced (Baffet et al., 2012). We suggest this may also be a consequence of damage.

One interesting feature of the damage-induced phenotype is that some polarity factors are very rapidly lost from damaged cells, whereas others are not. The damage is induced during the course of fixation, leaving very little time for soluble cytoplasmic proteins to wash out of the cell. The proteins that disappear, such as aPKC and Dlg, show a robust and stable localisation to the apical and lateral cortex respectively in the absence of damage. This suggests that they are only loosely associated with the cortex, and their steady state localisation is the result of a dynamic equilibrium between continuous association or delivery to the cortex and rapid release.

Although AMPK is not required for follicle cell polarity, it does play a role in the regulation of follicle cell size, as *ampkα*^3^ mutant cells show an increase in mean cell area even under normal conditions. This finding was unexpected as AMPK functions as sensor of energy availability and is responsible for regulating numerous metabolic processes under low energy conditions (reviewed by Hardie et al., 2012). Since AMPK restricts follicle cell growth even in well-fed flies, the kinase must be active under these conditions. AMPK is allosterically regulated by the binding of AMP, which accumulates as ATP levels fall and the ATP/ADP ratio goes down. The activity of AMPK therefore suggests that the follicle cells are under a weak, constitutive energetic stress that maintains tight coupling between energy availability and cell growth.

*ampkα*^3^ mutant cells in starved egg chambers are also increased in size relative to their wild-type neighbours. As AMPK is activated under low-energy conditions, we expected the difference in cell size to be greater in starved flies than in healthy ones. Although no difference was found when comparing the cell size of well-fed and starved *ampkα*^3^ mutant cells at stage 4 and 7, we detected an increase in cell size at stage 9. Egg chamber development in *Drosophila* is coupled to nutrient availability, and egg production slows down under starvation conditions due to insufficient insulin signaling (Drummond-Barbosa and Spradling, 2001). The difference between starved and well-fed mutant cells may therefore only become apparent in late stage egg chambers that have passed this nutrient checkpoint.

AMPK probably exerts its growth inhibitory effect through the Tsc1/Tsc2 complex, which is activated by AMPK phosphorylation of Tsc2 (Inoki et al., 2003). Both Tsc1 and Tsc2 have been shown to regulate cell growth; mutation of either factor cell-autonomously promotes increased cell size in a variety of tissues by activating the TOR Complex 1 (TORC1) (Gao and Pan, 2001; Ito and Rubin, 1999; Potter et al., 2001; Sun et al., 2010; Tapon et al., 2001). It therefore seems likely that AMPK regulates cell growth in developing follicle cells by activating Tsc1/Tsc2 to inhibit TORC1, thereby balancing nutrient availability with tissue growth.

Science relies on careful and reliable experimental procedure. Here we present a story that highlights this basic precept. In the course of our work we identified a straightforward technical problem that produces an artefact that has been repeatedly mistaken for genuine results, leading to the publication of incorrect conclusions. Although these were inadvertent mistakes, we deeply regret the time and effort that others may have spent attempting to reproduce these results.

## Materials and Methods

### *Drosophila* stocks

The following stocks were used in this study: *FRT101 ampkα^1^* (Mirouse et al., 2007), *FRT101 ampkα^3^* (Mirouse et al., 2007), *FRTG13 Dg^O86^* (Christoforou et al., 2008), *FRT82B pak^14^* (Newsome et al., 2000), *FRT101* (1844, Bloomington Drosophila Stock Center), *FRTG13 Ubi-GFP.nls* (5826, Bloomington Drosophila Stock Center) balanced over CyO. *Ubi-mRFP.nls FRT80B* (30852, Bloomington Drosophila Stock Center). The MARCM stock was generated as previously described (Wu and Luo, 2006) using *UAS-mCD8GFP.LL4* (5136, Bloomington Drosophila Stock Center), *Actin5C^NP1084^* (112494, Drosophila Genetic Resource Center) and *hsFLP1* (6, Bloomington Drosophila Stock Center) on the X and *FRTG13 tubP-GAL80.LL2* (5140, Bloomington Drosophila Stock Center) on 2R.

### *Drosophila* methods

Starvation conditions, somatic clone induction, immunofluorescence and cell imaging were performed as previously described (Mirouse et al., 2007; Mirouse et al., 2009).

### Imaging and cell measurements

Confocal micrographs were collected on an Olympus FV1000 inverted microscope. Anti-Dlg immunofluorescence signal was used to mark cell outlines. The area of individual cells was determined by taking an optical section through the layer of follicle cells facing the cover slip and measuring the area using ImageJ. GFP-negative mutant cells (*ampkα^3^*/*ampkα^3^*) denoted as “*ampkα^3^*” were compared to GFP-positive control cells with at least one WT copy of *ampk* (*ampkα^3^*/+ and *+/+*) denoted as “*ampkα*^3^/+”.

### Reagents

The following antibodies were used in this study: rabbit anti-aPKC (1/200, Santa Cruz), mouse anti-Crumbs (1/25), mouse anti-Cora (1/200), mouse anti-Armadillo (1/50), mouse anti-α-spectrin (1/100), and mouse anti-Dlg (1/50) (all from Developmental Studies Hybridoma Bank), mouse anti-FasIII (1/100, gift from C. Goodman), rabbit anti-βH-spectrin (1/100) (gift from D. Branton), rabbit anti-Baz N-term (1/1000, gift from A. Wodarz), rabbit anti-Par-6 (1/500, gift from D. Montell), rabbit anti-Dg (1/200, gift from W.-M. Deng), chicken anti-GFP (1/1000, Abcam). Rhodamine-Phalloidin was purchased from Invitrogen. Vectashield with DAPI was purchased from Vector Labs. Conjugated secondary antibodies were purchased from Jackson Immunoresearch and Invitrogen. 2-Deoxy-D-glucose, Berberine chloride form, Phenformin hydrochloride, Metformin hydrochloride, Paraquat dichloride, Linezolid, Tetracylcine, Chloramphenicol were purchased from Sigma–Aldrich and Oligomycin from Merck.

### Sequencing of mutant alleles

gDNA was extracted from heterozygous mutant females using ReliaPrep^TM^ gDNA Tissue Miniprep System (Promega). Regions spanning the mutant lesions were amplified by PCR and sequenced using the following primers:

*ampk* alleles forward ATATTGTGAAGCACGGCAAG

*ampk* alleles reverse GGAGGTCCTTTTGGAACCAC

*Dg^O86^* allele forward AATTGAGCCAAAGGATGTGC

*Dg^O86^* allele reverse CTTCATTTGGTGGGCAATCT

Sequence tracks were assembled using LaserGene SeqMan Pro (DNASTAR).

## Note added in proof

In light of the results reported here, two papers by Mirouse et al. have been retracted (Mirouse et al., 2013; Mirouse et al., 2009).

## Supplementary Material

Supplementary Material
