# Supplementary material for: Damage to the Drosophila follicle cell epithelium produces “false clones” with apparent polarity phenotypes
Source: Biol Open. 2013 Sep 23;2(12):1313–20. doi: 10.1242/bio.20134671 (PMC3863415; doi:10.1242/bio.20134671)
Supplement: Supplementary Material [file supp_2_12_1313__index.html]

Damage to the Drosophila follicle cell epithelium produces “false clones” with apparent polarity phenotypes — Damage to the Drosophila follicle cell epithelium produces “false clones” with apparent polarity phenotypes — Supplementary Material 

# Damage to the *Drosophila* follicle cell epithelium produces “false clones” with apparent polarity phenotypes

## bio.20134671 Supplementary Material

**Files in this Data Supplement:**

- Supplementary Material - Timm Haack et al. doi: 10.1242/bio.20134671
