# Supplementary material for: Damage to the Drosophila follicle cell epithelium produces “false clones” with apparent polarity phenotypes
Source: Biol Open. 2013 Sep 23;2(12):1313–20. doi: 10.1242/bio.20134671 (PMC3863415; doi:10.1242/bio.20134671)
Supplement: Supplementary Material [file supp_bio.20134671_bio.20134671-s1.pdf]

Supplementary Material

Timm Haack et al. doi: 10.1242/bio.20134671

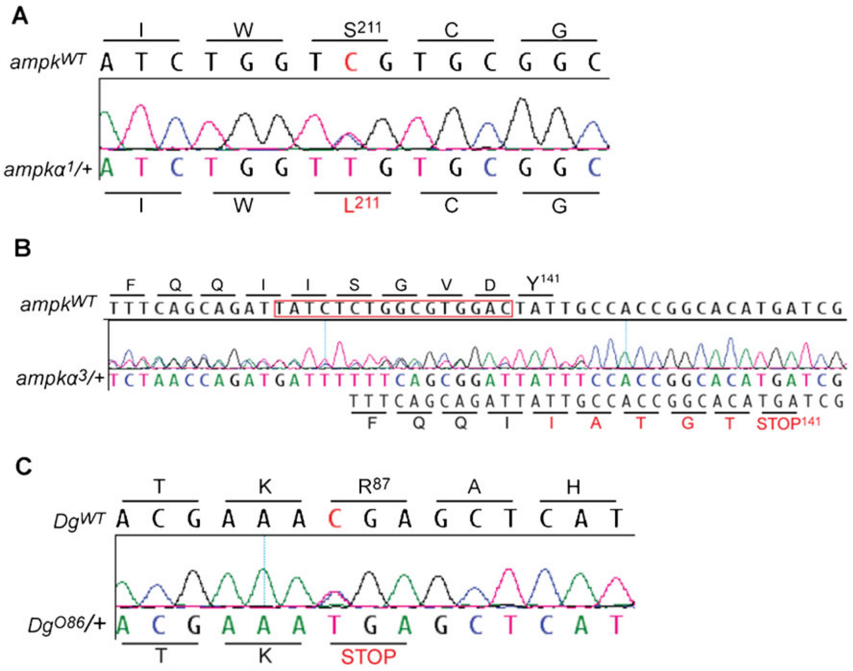

Fig. S1. Sequence verification of the *ampk* and *Dg* alleles used in this study. Genomic DNA was extracted from heterozygous mutant adult females, the mutant regions were amplified using PCR and sequenced to confirm the presence of the published mutations. (**A**) *ampkα*^{1} harbours a C to T mutation that changes the conserved serine 211 to leucine. (**B**) *ampkα*^{3} has a 16-bp deletion (red box) causing a premature STOP at position 141. (**C**) *Dg*^{O86} harbours a point mutation causing a premature STOP at position 87.

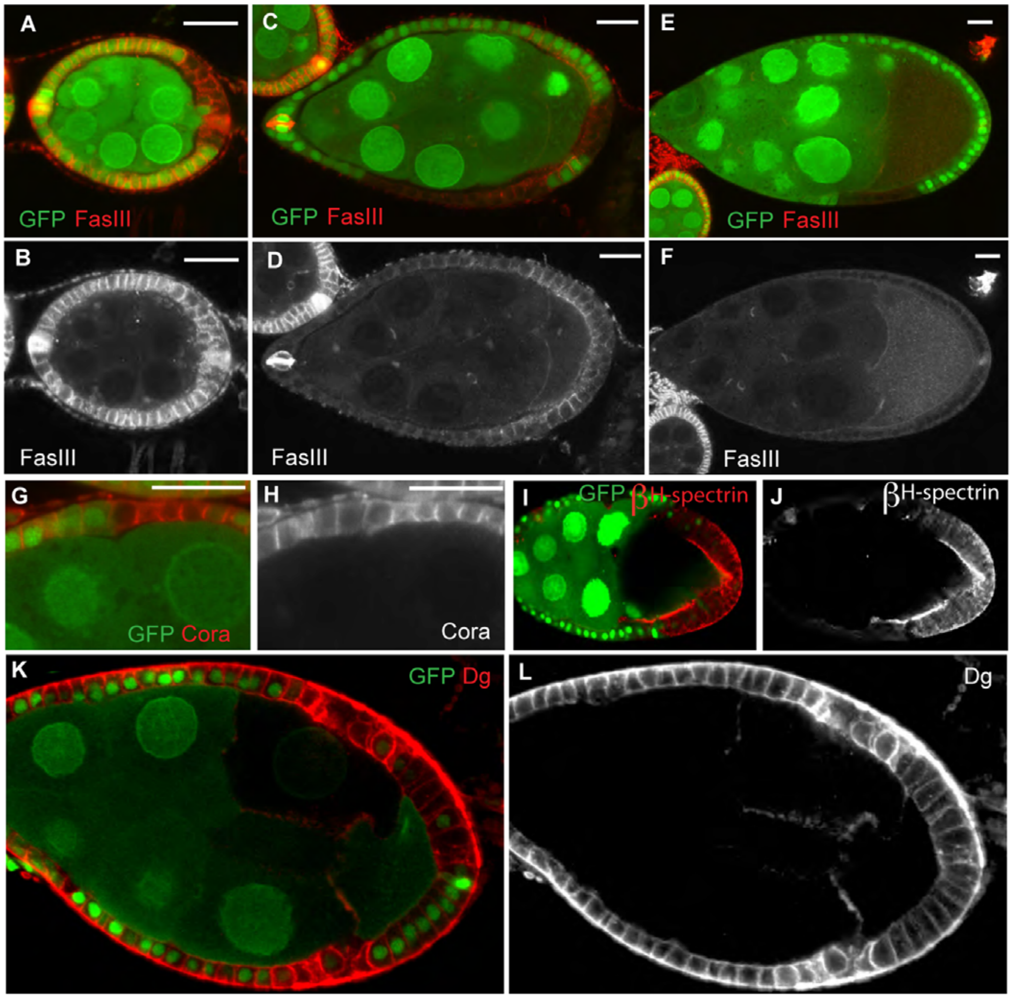

Fig. S2. FasIII, Coracle, βH-spectrin and Dystroglycan are unaffected in damage-induced false clones. Damaged egg chambers were deliberately produced using *FRTG13 GFPnls/CyO* females (**A–H**) or were identified in other experiments during the course of this study (**I–L**). Damaged cells are marked by the loss of GFP (green). (A–F) FasIII staining (red) at different stages of oogenesis. FasIII localises to the lateral cortex of the follicle cells in early stage egg chambers and is unaffected in damaged cells, even when the organisation of the epithelium is disrupted (A,B). A postmitotic stage 8 egg chamber in which the FasIII signal is restricted to the posterior follicle cells and the polar cells (C,D). A posterior false clone shows a slight reduction in the FasIII signal (C). FasIII has disappeared by stage 9 (E,F). (G,H) Coracle staining (red and panel H) in a stage 7 egg chamber containing a damage-induced false clone. Coracle remains at the lateral cortex in damaged cells. (I,J) A large, posterior, damage-induced false clone marked by the loss of GFP (green) stained for βH-spectrin (red). βH-spectrin staining is increased in the damaged area and extends basally. (K,L) A stage 8 egg chamber containing a damage-induced posterior clone stained for Dystroglycan (Dg, red and panel L). The Dg signal persists in the damaged cells and extends into the lateral and apical membrane domains. Scale bars: 20 µm.

Table S1. AMPK-activating drugs tested for enhancement of the low-energy polarity phenotype.

Feeding drugs in yeast paste 1d and 2d	Genotype	Loss of polarity in mutant clones
2-Deoxyglucose (2DG): 100 µM, 200 µM	Dg^{O86}	No
Berberine: 100 µM, 200 µM	Dg^{O86}, $ampk\alpha^{3}$	No
Oligomycin: 3 µM, 6 µM	Dg^{O86}	No
Phenformin: 10 mM, 20 mM	Dg^{O86}, $ampk\alpha^{3}$	No
Metformin: 25 mM, 50 mM, 100 mM	Dg^{O86}, $ampk\alpha^{1}$	No
Linezolid: 5 mM	Dg^{O86}	No
Tetracycline: 1 mM, 5 mM	Dg^{O86}	No
Chloramphenicol: 10 mM, 50 mM	Dg^{O86}	No
**Feeding drugs in instant dry food 1 d**		
Paraquat: 10 mM	Dg^{O86}, $ampk\alpha^{3}$	No
Berberine: 100 µM, 200 µM	Dg^{O86}	No
Oligomycin: 10 µM, 100 µM, 1000 µM	Dg^{O86}	No
Berberine: 100 µM + Paraquat 50 mM	Dg^{O86}	No
**Feeding drugs in low-glucose food 1 d**		
Berberine: 100 µM	Dg^{O86}	No
**Incubation of live egg chambers for 2 h at 25℃**		
2-Deoxyglucose (2DG): 50 mM	Dg^{O86}	No
Berberine: 100 µM	Dg^{O86}	No
Oligomycin: 100 µM	Dg^{O86}	No

Table S2. $ampk\alpha^{3}$ mutant cells show increase in cell size. Mean cell area with standard deviations (s.d.) are shown. Three stages of egg chamber development were analysed in well-fed (fed) and starved (ST) flies.

	stage 4 fed	stage 7 fed	stage 9 fed	stage 4 ST	stage 7 ST	stage 9 ST
$ampk\alpha^{3}$	18.41±4.90 µm^2	35.91±10.30 µm^2	66.29±18.81 µm^2	18.60±3.99 µm^2	37.50±9.62 µm^2	75.00±27.73 µm^2
$ampk\alpha^{3}$/+	15.35±3.66 µm^2	23.82±6.11 µm^2	46.07±9.18 µm^2	12.85±2.87 µm^2	24.88±3.90 µm^2	45.99±13.98 µm^2
FRT101	15.11±2.72 µm^2	23.86±8.00 µm^2	48.09±9.65 µm^2	12.26±3.32 µm^2	20.80±5.73 µm^2	54.10±7.30 µm^2
FRT101/+	14.93±2.76 µm^2	25.00±10.50 µm^2	46.57±9.98 µm^2	13.36±5.23 µm^2	19.93±4.65 µm^2	54.75±9.72 µm^2

Biology Open
